# Genome-wide identification of lncRNAs and mRNAs differentially expressed in non-functioning pituitary adenoma and construction of an lncRNA-mRNA co-expression network

**DOI:** 10.1242/bio.037127

**Published:** 2018-11-30

**Authors:** Weikang Xing, Zhenyu Qi, Cheng Huang, Nan Zhang, Wei Zhang, Yao Li, Minyan Qiu, Qi Fang, Guozhen Hui

**Affiliations:** 1 Department of Neurosurgery, The First People's Hospital of Wujiang District, Suzhou 215000, People's Republic of China; 2 Department of Neurosurgery, The First Affiliated Hospital of Soochow University, Suzhou 215000, People's Republic of China; 3 Department of Biology, McDaniel College, 2 College Hill, Westminster, MD 21157, USA; 4 Department of Neurology, The First Affiliated Hospital of Soochow University, Suzhou 215000, People's Republic of China; 5 State Key Laboratory of Genetic Engineering, Institute of Genetics, School of Life Science, Fudan University, Shanghai 200433, People's Republic of China

**Keywords:** Long non-coding RNA, Non-functioning pituitary adenomas, Microarray, Formalin-fixed and paraffin-embedded tissues, Co-expression network, Tumorigenesis

## Abstract

The involvement of long non-coding RNAs (lncRNAs) during tumorigenesis is a recent emerging theme. Yet no systematic evaluation of lncRNAs has been previously reported for non-functioning pituitary adenoma (NFPA), a fairly common type of intracranial tumor. Here, we report the first genome-wide expression profile for lncRNAs and mRNAs in NFPA, using formalin-fixed and paraffin-embedded tissue specimens. Using microarray analyses, we identified 113 lncRNAs and 80 mRNAs differentially expressed in NFPA; this list includes lncRNAs previously implicated in a variety of cancers. Using real-time quantitative reverse transcription polymerase chain reaction (qRT-PCR) we further confirmed differential expression in NFPA for ten of the 113 lncRNAs. Using these ten doubly confirmed lncRNAs, we constructed an lncRNA-mRNA co-expression network comprising of 130 specific lncRNA-mRNA co-expression relationships. In addition, we conducted GO and KEGG analyses for the 80 mRNAs differentially expressed in NFPA. Our microarray and qRT-PCR analyses provided a working list of lncRNAs that may be functionally relevant to NFPA tumorigenesis. Our co-expression network in turn connected these largely uncharacterized lncRNAs to specific mRNAs, whose roles we further elucidated via GO and KEGG analyses, thus providing specific, testable hypotheses for the functions of these lncRNAs. Together, our study laid the foundation for future investigation of the specific function and mechanism by which lncRNAs are involved in NFPA tumorigenesis.

## Abstract (precis)

**Summary statement:** We identified long non-coding RNAs and mRNAs differentially expressed in non-functioning pituitary adenomas via microarray analyses, and provided working hypotheses for how these RNAs may function via co-expression network analyses.

## INTRODUCTION

Pituitary adenomas (PAs) account for approximately 15% of intracranial primary tumors with a prevalence of close to one case per 1000 individuals (Daly et al., 2006; Fernandez et al., 2010). Approximately 30% of all PAs are considered non-functioning pituitary adenomas (NFPAs); NFPAs as a group gains its distinction because these tumors do not secret excessive pituitary hormones (Katznelson et al., 1993). As such, NFPAs do not cause, and therefore cannot be diagnosed by, clinical hormone hypersecretion. Instead they are usually not diagnosed until these neoplasms grow rather large in size and begin to locally compress intracranial nerves and brain tissues, leading to symptoms. Despite a consistent level of research interest, our understanding of the molecular mechanisms that cause NFPA remains poor. This is partly evidenced by the fact that no clearly effective medications are currently available for NFPA, and that no molecular markers are currently established to diagnose NFPA before neurological symptoms occur. Identification of molecular players for NFPA tumorigenesis thus appears both critical and timely.

The need to identify molecular players for NFPA tumorigenesis coincides with the very recent recognition of a class of molecular players involved in tumorigenesis: long non-coding RNAs (lncRNAs). This class of non-coding RNAs (ncRNAs) longer than 200 nucleotides (Gibb et al., 2011) have been implicated in the regulation of gene expression at the epigenetic, transcriptional, or post-transcriptional level (Caley et al., 2010), even though they do not encode any protein products themselves. Regulation of gene expression by lncRNAs in turn affects critical cellular decisions such as cell division (Guttman et al., 2011) and apoptosis (Braconi et al., 2011), immediately raising the possibility of their involvement in tumorigenesis. Indeed, a number of lncRNAs are now established molecular markers of tumorigenesis and are subjects of intense investigation. For example, the well-known HOX antisense intergenic RNA (HOTAIR) has been shown to interact with polycomb repressive complex 2 (PRC2), alter H3 lysine 27 (*H3K27*) methylation and therefore gene expression, over-express in breast cancer and enhance tumor invasiveness and metastasis (Gupta et al., 2010). For another example, metastasis associated lung adenocarcinoma transcript 1 (*MALAT1*) has been shown to interact with serine/arginine (SR) proteins (Tripathi et al., 2010), modulate mRNA alternative splicing (Tripathi et al., 2010), and over-express in non-small-cell lung cancer (Ji et al., 2003), hepatocellular carcinomas (Lin et al., 2007) and breast cancer (Guffanti et al., 2009). Consistent with their involvement in tumorigenesis, lncRNAs are differentially expressed in many neoplasms such as glioblastoma multiforme (GBM, Yan et al., 2015), papillary thyroid carcinoma (Lan et al., 2015), lung adenocarcinoma (Wang et al., 2015) and colorectal cancer (Xue et al., 2015). However, the expression of lncRNAs in NFPA has not been systematically evaluated to date.

The evident need to construct a genome-wide expression profile for lncRNAs in NFPA in order to identify molecular players for NFPA tumorigenesis mandates that normal pituitary (NP) be available as the necessary control. However, the essential function of pituitary means that fresh-frozen NP tissue is rarely available. Most NP tissues available are in the form of formalin-fixed and paraffin-embedded (FFPE) specimens. While FFPE specimens were historically unpopular for RNA analyses due to the concern of possible RNA fragmentation, recent advances in RNA extraction and microarray technology for FFPE specimens have now made it possible to explore the rich information contained within FFPE specimens (Liu and Xu, 2011; Lu et al., 2011; Ludyga et al., 2012; Morton et al., 2014).

In this study, we report the first genome-wide profiling of lncRNAs in NFPA using FFPE specimens, in an effort to identify potential molecular players for NFPA tumorigenesis. In addition to identifying lncRNAs differentially expressed in NFPA, we also identified mRNAs differentially expressed in NFPA. We did this because a) genome-wide profiling of mRNAs in NFPA has also not been reported before, b) differentially expressed mRNAs also represent potential molecular players for NFPA tumorigenesis and c) the much better understood mRNAs can serve as a link between lncRNAs and their largely unexplored functional assignments. We hence constructed a co-expression network that connects the largely uncharacterized lncRNAs to specific mRNAs, whose roles we further elucidated via Gene Ontology (GO) analyses and Kyoto Encyclopedia of Genes and Genomes (KEGG) analyses.

## RESULTS

### Identification of lncRNAs and mRNAs differentially expressed in NFPA

**Fig. 1.**
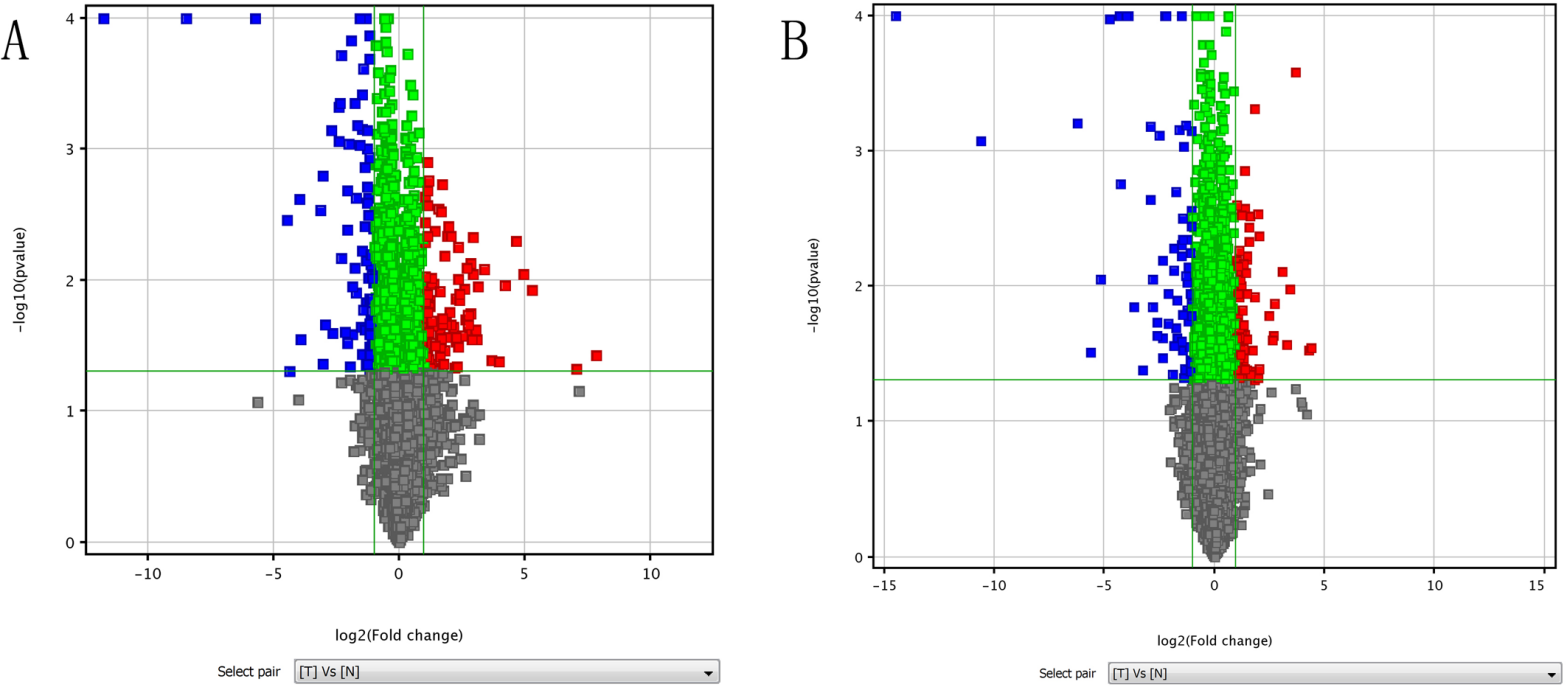
**Identification of lncRNAs and mRNAs differentially expressed in NFPA.** The expression profiles of lncRNAs and mRNAs were compared between non-functioning pituitary adenoma (NFPA) and normal pituitary (NP) using formalin-fixed and paraffin-embedded (FFPE) tissue specimens. Volcano plot of lncRNAs (A) and mRNAs (B) illustrate the change in the expression level of each transcript (represented as a single square), when its expression in NFPA is compared to its respective expression in NP. The horizontal axis represents fold of change in expression (on a log_2_ scale); the green vertical line on the left thus represents a twofold downregulation, and the green vertical line on the right represents a twofold upregulation. The vertical axis represents *P* value (on a negative log_10_ scale); the green horizontal line thus represents a *P* value of 0.05. The red squares thus represent transcripts significantly upregulated in NFPA, and the blue squares represent transcripts significantly downregulated in NFPA, based on our statistical criteria.

**Fig. 2.**
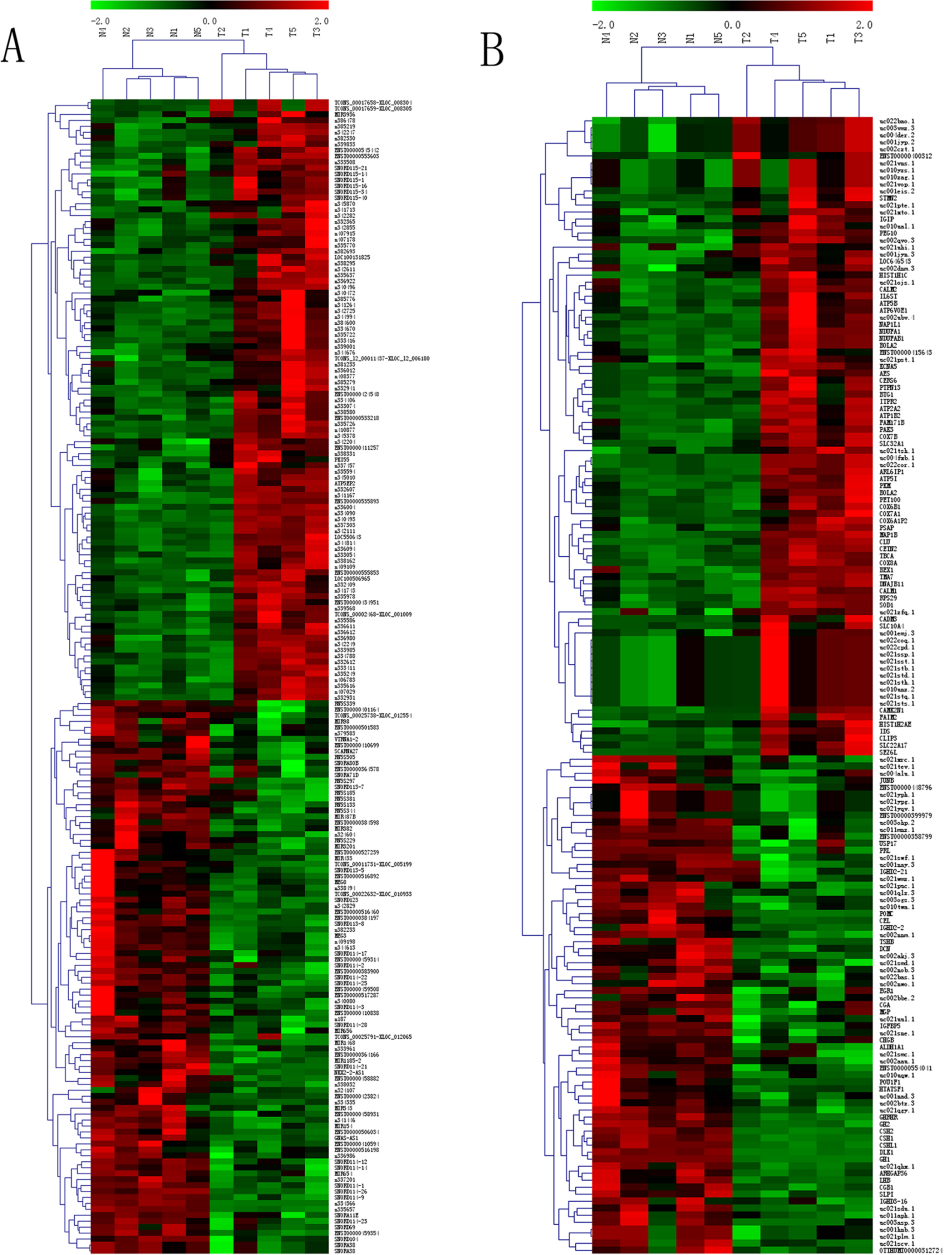
**Hierarchical clustering analyses of differentially expressed lncRNAs and mRNAs.** Hierarchical clustering analyses were used to arrange FFPE samples into groups according to the expression levels of differentially expressed lncRNAs (A) and mRNAs (B). N: NP. T: NFPA. Red: high relative expression. Green: low relative expression.

We aimed to construct a genome-wide expression profile for NFPA involving both lncRNAs and mRNAs, and to determine which lncRNAs and mRNAs are expressed in NFPA in a different manner compared to their respective expression in normal pituitary. To this end, we performed microarray analyses comparing five NFPA samples and five NP samples, randomly selected from a collection of 42 FFPE tissue samples. We detected a total of 30,020 lncRNAs and 25,994 mRNAs. Compared to NP, 87 lncRNAs (red squares in Fig. 1A) and 54 mRNAs (red squares in Fig. 1B) were significantly (*P*<0.05) upregulated (≥twofold difference) in NFPA. Using identical statistical criteria, we determined that 26 lncRNAs (blue squares in Fig. 1A) and 26 mRNAs (blues squares in Fig. 1B) were downregulated in NFPA. The identities of the 193 differentially expressed transcripts identified herein are listed in Table S1. We conducted hierarchical clustering analyses using these 193 differentially expressed transcripts, which classified the 10 FFPE samples into two groups: NP (‘N’ in Fig. 2A and B), and NFPA (‘T’ in Fig. 2A and B), as expected.

### Validation of differentially expressed lncRNAs using real time quantitative reverse transcription polymerase chain reaction (qRT-PCR)

**Fig. 3.**
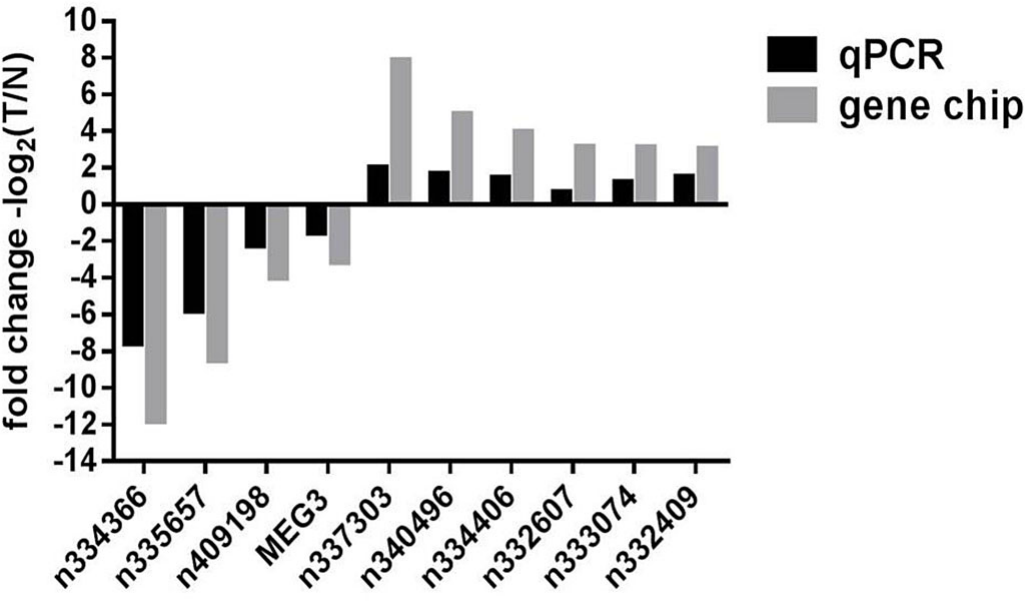
**Validation of differentially expressed lncRNAs using qRT-PCR.** The expression levels of ten individual lncRNAs were compared between non-functioning pituitary adenoma (NFPA) and normal pituitary (NP) using formalin-fixed and paraffin-embedded (FFPE) tissue specimens. The change in the expression level of each lncRNA, when its expression in NFPA is compared to its respective expression in NP, is illustrated as a vertical bar. The vertical axis represents fold of change in expression (on a log_2_ scale). Black bars represent qRT-PCR results, which validated microarray results (gray bars).

To investigate the validity of our microarray results, we used qRT-PCR to evaluate the expression levels of individual transcripts identified by microarrays. Since our particular interest lies in lncRNAs, and since microarrays have long been established as a mainstream approach to evaluating mRNA expressions (Mueller et al., 2010), we focused on lncRNA validations in this study. From the 20 lncRNAs that exhibited the greatest expression fold changes per microarrays (Table S1), we randomly selected four downregulated lncRNAs (*n334366*, *n335657*, *n409198*, *MEG3*) and six upregulated lncRNAs (*n337303*, *n340496*, *n334406*, *n332607*, *n333074*, *n332409*) for qRT-PCR analyses. Using qRT-PCR, we evaluated their expression levels in the remaining 32 FFPE tissue samples from our initial collection (see 2.1, 30 NFPAs and 2 NPs). qRT-PCR results demonstrated that *n334366*, *n335657*, *n409198* and *MEG3* were downregulated in NFPA, and that *n337303*, *n340496*, *n334406*, *n332607*, *n333074*, and *n332409* were upregulated in NFPA (Fig. 3, black bars), which were 100% concordant with the microarray results (Fig. 3, gray bars). These qRT-PCR results thus directly support the validity of these ten differentially expressed lncRNAs that we identified via microarrays, and indirectly support the validity of other differentially expressed transcripts (Table S1) that we identified via microarrays.

### Construction of a lncRNA-mRNA co-expression network

The direct validation (Fig. 3) of the ten lncRNAs that we identified to be differentially expressed in NFPA (all with a fold change>8 per microarrays; Table S1; Fig. 3) suggested functional involvement of these lncRNAs in NFPA pathobiology. However, no functional studies have been reported for the majority of these ten lncRNAs. To begin to explore the potential function of these ten lncRNAs, we attempted to construct a lncRNA-mRNA co-expression network, an approach that has become widely utilized and accepted (Li et al., 2013; Gu et al., 2015; Wang et al., 2015). The established principle of this methodology is that, if the expression levels of two transcripts (in this case, a single lncRNA and a single mRNA) are consistently linearly correlated across, not two, but many samples (in this case, ten NFPA or NP samples, see 2.1), these two transcripts are thereby determined to be co-expressed. Such co-expression may suggest that the expression of these two transcripts is either co-regulated by a common regulator(s), or that one transcript regulates the expression of the other. In particular, since the most prominent theme of lncRNA function, based on our current understanding, appears to be regulation of mRNA expression (Panda et al., 2017), co-expression between an lncRNA and a specific mRNA may indeed provide the first hint that this lncRNA regulates the expression of this mRNA. Whether the relationship is co-regulatory or cross-regulatory, since mRNA functions are in general much better understood than lncRNA functions, identifying co-expression relationships between an lncRNA and a specific mRNA may begin to reveal the function and/or mechanism of action of that lncRNA.

**Fig. 4.**
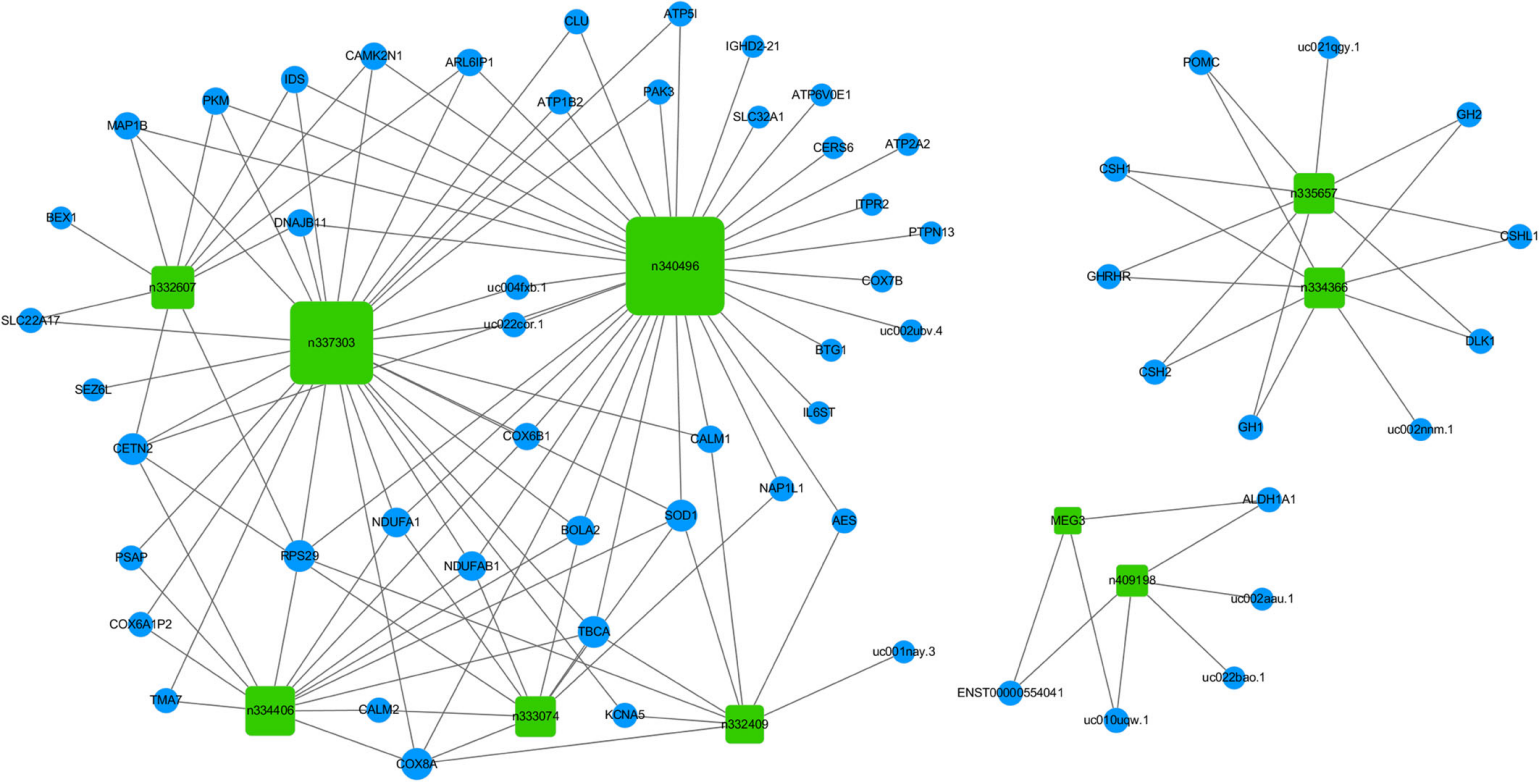
**An lncRNA-mRNA co-expression network.** Ten lncRNAs (green rectangles) that are differentially expressed in NFPA identified via microarrays and verified via qRT-PCR, are connected to their respective co-expressed (|PCC|>0.9, *P*<0.0005) mRNAs (blue circles) with lines.

To construct such an lncRNA-mRNA co-expression network, we first aimed to identify mRNAs that are co-expressed with any of the ten lncRNAs (Fig. 3) in a highly significant manner. To this end, we investigated whether any of the 80 mRNAs differentially expressed in NFPA per microarrays (Fig. 1B, blue and red squares) are co-expressed with any of these ten lncRNAs. For each lncRNA-mRNA pair (800 total pairs examined), we evaluated whether the expression levels of the lncRNA and the mRNA are consistently linearly correlated across all ten NFPA or NP samples (see 2.1) by calculating the Pearson Correlation Coefficient (PCC, Prieto et al., 2008), which has been widely used to measure linear correlation therefore co-expression (Li et al., 2013; Gu et al., 2015; Wang et al., 2015). In general, a PCC value of 1 indicates a perfect, positive linear correlation. A PCC value of −1 indicates a perfect, negative linear correlation. A PCC value of 0 indicates no linear correlation. We employed extremely stringent criteria (Li et al., 2013) for correlation (PCC>0.9 for positive correlation or PCC<−0.9 for negative correlation) and for statistical significance (*P*<0.0005, Table S2). Based on such criteria, we identified a total of 59 mRNAs (Table S2) each of which is co-expressed with at least one of the ten lncRNAs. We then constructed the lncRNA-mRNA co-expression network (Fig. 4) to visually represent the complex co-expression relationships (Fig. 4, lines, totaling 130) among the ten lncRNAs (Fig. 4, green squares) and 59 mRNAs (Fig. 4, blue circles). Interestingly, of all the co-expression relationships (Fig. 4, lines), only three are negative correlations (*IGHD2-21* and *n340496*, *uc022bao.1* and *n409198*, *uc001nay.3* and *n332409*). lncRNA *n340496* is co-expressed with the greatest number (35) of the 80 mRNAs investigated, while mRNA *TBCA* is co-expressed with the greatest number (five) of the ten lncRNAs investigated (Fig. 4; Table S2).

### GO analysis and KEGG pathway analysis of mRNAs differentially expressed in NFPA

We set out to determine the major functional themes of the 80 mRNAs differentially expressed in NFPA per microarrays (Fig. 1B, blue and red squares), reasoning that such themes will shed light on NFPA tumorigenesis. In addition, since 59 of these 80 mRNAs are co-expressed (Fig. 4) with at least one of the ten lncRNAs (Fig. 3), determination of the functional themes of such mRNAs will potentially contribute to our understanding of these ten lncRNAs as well. To accomplish these goals, we performed GO analysis (Ashburner et al., 2000) as well as KEGG pathway analysis (Ogata et al., 1999).

**Fig. 5.**
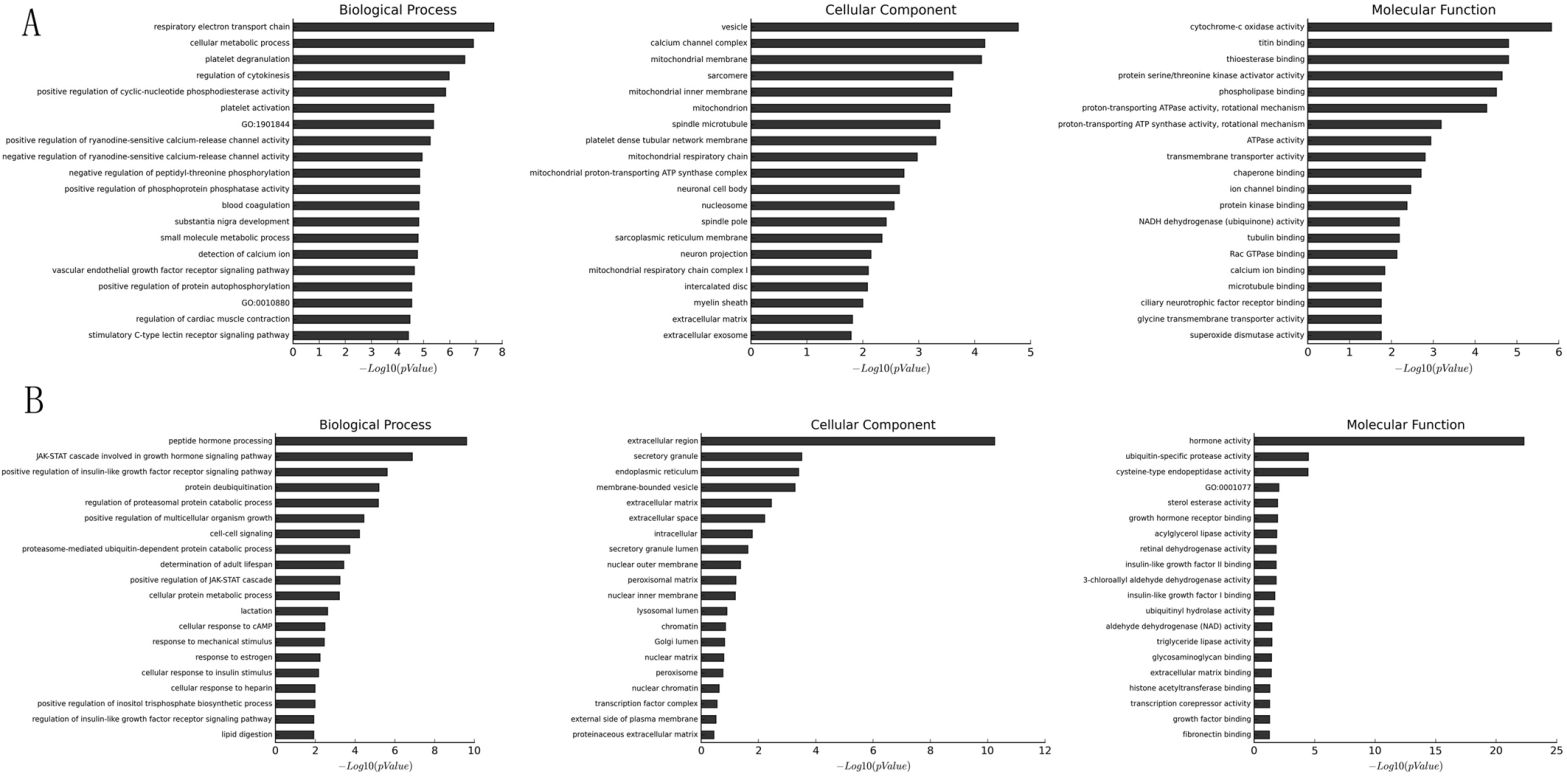
**Gene Ontology (GO) analysis of mRNAs differentially expressed in NFPA.** The 20 most enriched GO terms (vertical axis) for mRNAs upregulated (A) or downregulated (B) in NFPA are arranged based on −log_10_(*P* value) (horizontal axis). The most enriched GO term for biological process (left), cellular component (middle) and molecular function (right) is at the top of the vertical axis.

GO analysis is widely used to determine the emerging themes among a group of gene products along three dimensions: biological process, cellular component and molecular function (Li et al., 2013; Lan et al., 2015; Wang et al., 2015). For mRNAs upregulated in NFPA per microarrays (Fig. 1B, red squares), the most significant (as determined by *P* values) emerging themes are ‘respiratory electron transport chain’ (biological process, Fig. 5A, left), ‘vesicle’ (cellular component, Fig. 5A, middle), and ‘cytochrome-c oxidase activity’ (molecular function, Fig. 5A, right). For mRNAs downregulated in NFPA per microarrays (Fig. 1B, blue squares), the most significant emerging themes are ‘peptide hormone processing’ (biological process, Fig. 5B, left), ‘extracellular region’ (cellular component, Fig. 5B, middle) and ‘hormone activity’ (molecular function, Fig. 5B, right). The complete GO analysis results can be found in Table S3.

**Fig. 6.**
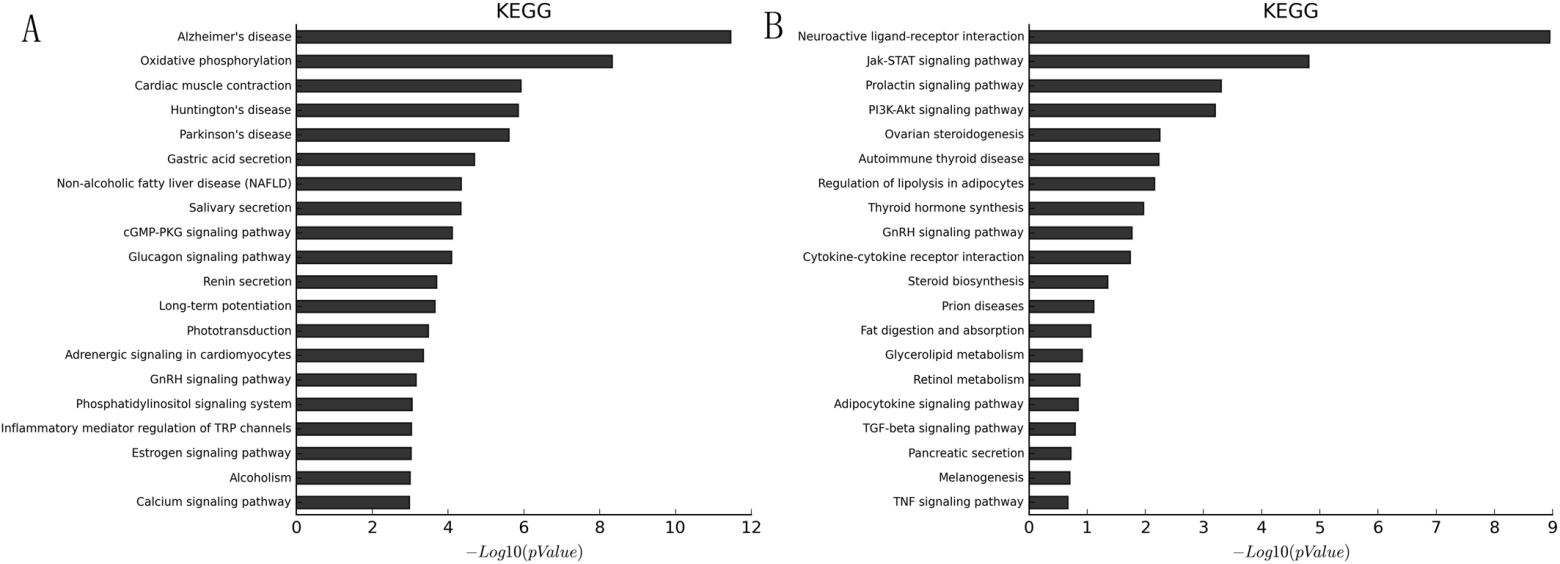
**Kyoto Encyclopedia of Genes and Genomes (KEGG) pathway analysis of mRNAs differentially expressed in NFPA.** The 20 most enriched KEGG pathways (vertical axis) for mRNAs upregulated (A) or downregulated (B) in NFPA are arranged based on −log_10_(*P* value) (horizontal axis). The most enriched KEGG pathway is at the top of the vertical axis.

KEGG pathway analysis is widely used to determine emerging biological pathways among a group of gene products (Li et al., 2013; Lan et al., 2015; Wang et al., 2015). For mRNAs upregulated in NFPA per microarrays (Fig. 1B, red squares), the most significant (as determined by *P* values) emerging biological pathway is ‘Alzheimer's disease’ (Fig. 6A); 12 genes in this pathway were upregulated in NFPA per microarrays. For mRNAs downregulated in NFPA per microarrays (Fig. 1B, blue squares), the most significant emerging biological pathway is ‘neuroactive ligand-receptor interaction’ (Fig. 6B); nine genes in this pathway were downregulated in NFPA per microarrays. The complete KEGG pathway analysis results can be found in Table S4.

## DISCUSSION

The involvement of lncRNAs during tumorigenesis is a recent emerging theme (Yoshimizu et al., 2008; Kim et al., 2013). Similar to classical oncogenes or tumor-suppressor genes such as *eIF4F* (*eukaryotic translation initiation factor 4F*, Bhat et al., 2015) and *DLK1* (*Delta-like 1 homolog*, Kawakami et al., 2006), lncRNAs may act as oncogenes or tumor-suppressor genes by altering gene expression (Zhou et al., 2012; Kim et al., 2013). Such involvement of lncRNAs during tumorigenesis thus highlights the urgency of identifying specific lncRNAs differentially expressed in each human cancer. While genome-wide lncRNA expression profiles have been established for a number of human tumors such as osteosarcoma (Li et al., 2013), lung adenocarcinoma (Wang et al., 2015) and colorectal cancer (Xue et al., 2015), our work represents the first report of a genome-wide lncRNA expression profile for NFPA.

Here, we report the identification via microarray analyses of 113 lncRNAs (and 80 mRNAs) that are differentially expressed in NFPA when compared to NP (fold-change≥2, *P*<0.05; Fig. 1; Table S1). Our subsequent qRT-PCR analyses using an expanded number of samples further supported the validity of such identification (Fig. 3). While these identifications were made using FFPE samples, and not fresh-frozen samples, recent advances in RNA extraction technology began to address historical concerns of using FFPE samples (Farragher et al., 2008) and FFPE samples are now considered reliable for mRNA (Liu and Xu, 2011; Lu et al., 2011; Ludyga et al., 2012) and lncRNA (Morton et al., 2014) studies.

Of the 113 lncRNAs differentially expressed in NFPA that we have identified, some have already been established as prominent tumorigenesis players, one example of which is *MEG3*. *MEG3* is an imprinted gene located on chromosome 14q32.3 (Miyoshi et al., 2000). It has been reported to function as a critical tumor-suppressor via both *p53*-dependent and *p53*-independent pathways (Zhou et al., 2012). Consistent with its tumor-suppressor role, the reduction of *MEG3* expression has been observed in NFPA (Gejman et al., 2008; Cheunsuchon et al., 2011) as well as other types of tumors such as hepatocellular carcinoma (Braconi et al., 2011), non-small-cell lung cancer (Lu et al., 2013), glioma (Wang et al., 2012) and meningioma (Zhang et al., 2010). Importantly, our study here confirmed this reduction of *MEG3* expression in NFPA: our microarray analyses of ten FFPE samples indicated that *MEG3* expression was downregulated almost ninefold in NFPA (Fig. 3; Table S1), and our qRT-PCR analyses of 32 FFPE samples indicated that *MEG3* expression was downregulated almost 2.5-fold in NFPA (Fig. 3). A more recently revealed, therefore less well-known, lncRNA implicated in tumorigenesis is *ENST00000501583*. *ENST00000501583* expression has been reported to be downregulated in hepatocellular carcinoma (Liu et al., 2014), raising the possibility that *ENST00000501583* may function as a tumor suppressor. Importantly, our study here revealed the reduction of *ENST00000501583* expression in another type of tumor – NFPA; our microarray analyses indicated that *ENST00000501583* expression was downregulated by more than fourfold in NFPA (Table S1). Both *MEG3* and *ENST00000501583* are among the ten most significantly downregulated lncRNAs in NFPA (Table S1). Since investigation of lncRNAs in general is still at its early stage, the function of the majority of any lncRNAs is unknown. Likewise, the function of the majority of the 113 lncRNAs we identified here is also unknown. However, the fact that known or emerging tumor suppressors are among the most differentially expressed lncRNAs within this group of 113 lncRNAs provides additional support to the functional relevance of this group of 113 lncRNAs to NFPA, particularly for those with a greater fold of change in expression.

Since the function of the majority of any lncRNAs is unknown, much effort has been made to construct lncRNA-mRNA co-expression networks (Li et al., 2013; Fu et al., 2015; Gu et al., 2015; Wang et al., 2015). The rationale is that the establishment of a co-expression relationship between a specific lncRNA and a specific mRNA will lead to specific, experimentally testable hypotheses regarding the function of an lncRNA, since function of mRNAs is in general much better understood than that of lncRNAs. With this rationale in mind, we constructed an lncRNA-mRNA co-expression network. We focused on ten specific lncRNAs (Fig. 3) when we constructed this co-expression network, because these lncRNAs have been validated to be differentially expressed in NFPA by both microarrays (Table S1) and qRT-PCR (Fig. 3), and because they have a greater fold of change in expression (>eightfold per microarrays; Table S1). Our co-expression network, constructed by using extremely stringent criteria (see 2.3), revealed a total of 130 specific lncRNA-mRNA co-expression relationships (Fig. 4, lines). As rationalized, these co-expression relationships in turn open doors to specific, experimentally testable hypotheses regarding the function of these ten lncRNAs. For example, *n334366* is the most significantly downregulated lncRNA in NFPA (>3500-fold per microarrays; Table S1), and yet there is no report of its function in the literature. However, our co-expression network revealed that its expression is positively correlated with eight mRNAs including the well-studied *DLK1*. *DLK1* was previously reported to be downregulated in NFPA (Moreno et al., 2005; Butz et al., 2011), consistent with our study (Table S1). Furthermore, *DLK1* expression is regulated by the Notch pathway (Falix et al., 2012), which has been implicated in NFPA tumorigenesis (Moreno et al., 2005). Thus, three different hypotheses immediately emerge. 1) *n334366* positively regulates the expression of *DLK1*. 2) *DLK1* positively regulates the expression of *n334366*. 3) The Notch pathway regulates the expression of *n334366*. All three hypotheses can be readily tested in tissue culture cell systems or in model organisms, the results of which will undoubtedly advance our understanding of the function of lncRNA *n334366*. Thus, our study not only provided a working list of lncRNAs that may be functionally relevant to NFPA tumorigenesis, but it also provided working hypotheses regarding function of such lncRNAs that can be immediately tested.

While our main research focus is on lncRNAs, the example above illustrates how understanding of mRNAs differentially expressed in NFPA (80; Table S1) can greatly facilitate our understanding of lncRNAs. To this end, we conducted GO analyses and KEGG analyses for these 80 mRNAs differentially expressed in NFPA. These analyses take advantage of all 80 mRNAs and thus can provide insights and confidence beyond what single transcripts can provide individually. For example, KEGG analyses of downregulated mRNAs revealed Jak-STAT and PI3K-Akt signaling pathways as two of the most significantly represented pathways (Fig. 6B). This finding is not only consistent with well-established roles of Jak-STAT (Müller et al., 2005; Sansone and Bromberg, 2012) and PI3K-Akt (Bleau et al., 2009; Janku et al., 2012) in tumorigenesis in general, but is also consistent with recent findings that implicate Jak-STAT (Buslei et al., 2006) and PI3K-Akt (Rubinfeld and Shimon, 2012) specifically in pituitary tumors. The single most significantly represented pathway based on KEGG analyses of downregulated mRNAs is ‘neuroactive ligand-receptor interaction’ (Fig. 6B), represented by nine mRNAs (Table S4). This finding is not only consistent with reports that implicate γ aminobutyric acid (*GABA*, a neuroactive ligand) in pituitary tumors (End et al., 2005), but it also provides additional support to our lncRNA-mRNA co-expression network: five of the nine mRNAs are part of the co-expression network, and all five of them (*GH1*, *CSH2*, *GHRHR*, *SH1*, *GH2*) are disconnected from the majority of the co-expression network (Fig. 4, left), and are instead, part of a discrete ‘satellite’ co-expression network (Fig. 4, top right corner). Since KEGG analyses were conducted completely independently from co-expression network construction in terms of both methodology and procedure, the apparent correspondence between the neuroactive ligand-receptor interaction pathway (KEGG) and the discrete co-expression satellite argues for fundamental functional relevance of our lncRNA-mRNA co-expression network. Intriguingly, this co-expression satellite (Fig. 4, top right corner) not only includes five of nine members of the most significantly represented KEGG pathway, but it also includes the most significantly downregulated lncRNA *n334366* (>3500-fold). This newly revealed connection between *n334366* and the neuroactive ligand-receptor interaction pathway thus provided another biological context in which to investigate the function of lncRNA *n334366*. Since *n334366* was demonstrated to be the most significantly differentially expressed lncRNA in NFPA by both microarrays (Table S1) and qRT-PCR (Fig. 3), since *GH1*, *CSH2*, *GHRHR*, *CSH1* and *GH2* were demonstrated to function within the most significantly represented pathway of mRNAs downregulated in NFPA by KEGG analysis (Fig. 6B), and since *n334366*, *GH1*, *CSH2*, *GHRHR*, *CSH1* and *GH2* were demonstrated to be part of a discrete co-expression satellite disconnected from the majority of transcripts analyzed (Fig. 4), four distinct, independent analyses have thus impressively converged onto the same group of transcripts. This suggests that this co-expression satellite may represent one of the most critical groups of transcripts involved in NFPA tumorigenesis, and hence should be the focus of our imminent future research.

## CONCLUSIONS

To our knowledge, our study represents the first genome-wide lncRNA and mRNA profiling for NFPA. We have identified 113 lncRNAs and 80 mRNAs differentially expressed in NFPA using microarray analyses, this list includes lncRNAs that have been previously implicated in a variety of cancers. We have further confirmed differential expression in NFPA for ten of the 113 lncRNAs using qRT-PCR. Using these ten doubly confirmed lncRNAs, we constructed an lncRNA-mRNA co-expression network comprising 130 specific lncRNA-mRNA co-expression relationships. In addition, we conducted GO and KEGG analyses for the 80 mRNAs differentially expressed in NFPA. Our microarray and qRT-PCR analyses provided a working list of lncRNAs that may be functionally relevant to NFPA tumorigenesis. Our co-expression network in turn connected these largely uncharacterized lncRNAs to specific mRNAs, whose roles we further elucidated via GO and KEGG analyses, thus providing specific, testable hypotheses for the functions of these lncRNAs. In particular, the most differentially expressed lncRNA in NFPA, *n334366*, appears to form a distinct satellite in our co-expression network where its expression is correlated with many members of the neuroactive ligand-receptor interaction pathway, providing a potential first clue to the mechanism of action of this novel lncRNA. Together, our study laid the foundation for future investigation of the specific function and mechanism by which lncRNAs are involved in the pathobiology of NFPA.

## MATERIALS AND METHODS

### Non-functioning pituitary adenomas and normal pituitary samples

35 FFPE NFPA tissues were obtained from archived tissue samples derived from patients with NFPA and seven FFPE NP tissues were obtained from autopsy without PAs between July 2014 and May 2015 at the First Affiliated Hospital of Soochow University in China. Written informed consent was provided by patients' legal surrogates to permit use of abscised tissues. The study was approved by the Research Ethics Committee, the First Affiliated Hospital of Soochow University of China. The clinical data of participants (NFPA and NP) were listed in Table S5.

The number of specimens used for microarray analyses (five cancer specimens and five normal specimens) was determined based on standard practice in the field (Li et al., 2013; Lan et al., 2015; Wang et al., 2015; Xue et al., 2015; Yan et al., 2015). To ensure that our sampling was unbiased, we numbered all 35 NFPA specimens (1–35) and seven NP specimens (1–7), used a random number generator (random.org) to generate five random numbers for each group, and used the corresponding specimens for microarray analyses. The remaining 32 specimens were used for qRT-PCR validation.

### RNA extraction and purification

Total RNAs were extracted from FFPE tissue specimens by using RecoverAll™ Total Nucleic Acid Isolation Kit for FFPE (Ambion, USA) according to the manufacturer's protocol. Total RNAs were quantified with NanoDrop ND-2000 (Thermo Fisher Scientific). Total RNA yield averaged 7760 ng per sample (3564–15,700 ng), with a mean concentration of 193 ng/μl (89.1–392.5 ng/μl). Extracted total RNAs were further purified by RNeasy mini kit (Qiagen, Germany) according to the manufacturer's protocol.

### Gene microarray

GeneChip^®^ Human Transcriptome Array 2.0 (HTA2.0, Affymetrix, USA) was used for our study. The chip has been designed with approximately seven million specific probes, covering more than 245,000 coding and 40,000 non-coding RNA transcripts of the human genome. Each exon is covered by approximately ten probes, and each exon–exon boundary is covered by approximately four probes. Annotations of the transcripts were based on databases including RefSeq, Ensembl, UCSC, NONCODE, lncRNAdb, Vertebrate Genome Annotation (Vega), Mammalian Gene Collection (MGC), Broad Institute, Human Body Map lincRNAs, TUCP catalog and related literatures. Microarray hybridization, data acquisition and processing were performed by Shanghai OE Biotech Technology Co, Ltd (Shanghai, China).

### RNA labeling and microarray hybridization

Sample labeling, microarray hybridization and washing were performed based on the manufacturer's standard protocols of the Ambion^®^ WT Expression Kit (Ambion), WT Terminal Labeling and Controls Kit (Affymetrix) and GeneChip^®^ Hybridization, Wash, and Stain Kit (Affymetrix). Briefly, total RNAs were used to sequentially synthesize double-stranded cDNAs, cRNAs, and 2nd cycle cDNAs, Following fragmentation and biotin labeling, 2nd cycle cDNAs were hybridized onto the microarray. After washing and staining, the arrays were scanned by the Affymetrix Scanner 3000 (Affymetrix).

### Data acquisition and processing

The Affymetrix GeneChip Command Console (version 4.0, Affymetrix) software was used to extract raw expression data. Afterwards, the Expression Console (version1.3.1, Affymetrix) software was used to perform RMA normalization for both gene and exon level analyses. Genespring software (version 12.5, Agilent Technologies) was employed to finish the basic analysis. Differentially expressed genes with statistical significance between NFPA and NP samples were identified through Volcano plot filtering. The threshold set for upregulated and downregulated genes was a fold change ≥2.0 and a *P* value <0.05. Hierarchical clustering analysis was performed to reveal relationships among samples.

The microarray data presented in this study have been uploaded to Gene Expression Omnibus (GEO) database at the National Center for Biotechnology Information (NCBI) and the accession number is GSE77517.

### qRT-PCR

Total RNA from 32 FFPE samples (30 NFPAs and 2 NPs) was used for qRT-PCR to validate microarray results (A260/A280≥1.8 for RNA samples used). qRT-PCR was performed by using the SuperScript III First Strand Synthesis System (Invitrogen) and the ABI 7900HT Fast Real-Time PCR system per the manufacturers' protocols. The relative quantitative expressions of lncRNAs were calculated using the 2^−△△Ct^ method (Pfaffl, 2001). The primers used for qRT-PCR are listed in Table 1; *β-actin* (*ACTB*) was used as an internal control.

**Table 1.**
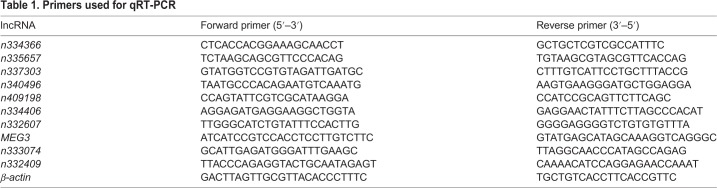
**Primers used for qRT-PCR**

### lncRNA-mRNA co-expression network

We constructed a lncRNA-mRNA co-expression network as follows for the ten lncRNAs (Fig. 3) doubly confirmed to be differentially expressed in NFPA by both microarray and qRT-PCR results.

We started with these ten lncRNAs and the 80 mRNAs (Fig. 1B, blue and red squares) differentially expressed in NFPA, and examined all possible lncRNA-mRNA pairs (800 pairs total).

For each RNA involved, we utilized its normalized expression value for each of the ten NFPA or NP samples. These values can be found in Table S1, under ‘Normalized Intensity’. For each lncRNA-mRNA pair, we then evaluated whether the normalized expression values of the lncRNA and the mRNA are consistently linearly correlated across all ten NFPA or NP samples by calculating the Pearson Correlation Coefficient (PCC) (Prieto et al., 2008). If |PCC| was greater than 0.9, and the *P* value was less than 0.0005, we considered this lncRNA-mRNA pair to be linearly correlated hence co-expressed. We identified 130 pairs that met these criteria out of the possible 800 pairs of lncRNA-mRNA. These 130 co-expression relationships, involving all ten lncRNAs and 59 of the possible 80 mRNAs, were then visually represented as the co-expression network using Cytoscape (http://www.cytoscape.org).

### GO analysis and KEGG pathway analysis of mRNAs differentially expressed in NFPA

The 54 upregulated mRNAs and 26 downregulated mRNAs were analyzed according to GO function categories (http://www.geneontology.org) and KEGG database (http://www.genome.jp/kegg), respectively.

### Statistical analysis

All statistical analyses were performed using the SPSS version 17.0 software (SPSS, Inc., Chicago, USA). Student's *t*-tests were performed to generate *P* values.

## Supplementary Material

Supplementary information
