# Supplementary material for: Genome-wide identification of lncRNAs and mRNAs differentially expressed in non-functioning pituitary adenoma and construction of an lncRNA-mRNA co-expression network
Source: Biol Open. 2018 Nov 30;8(1):bio037127. doi: 10.1242/bio.037127 (PMC6361197; doi:10.1242/bio.037127)
Supplement: Supplementary information [file biolopen-8-037127-s1.pdf]

Table S1. lncRNAs and mRNAs differentially expressed in non-functioning pituitary adenoma

Click here to Download Table S1

Table S2. PCC values of co-expressed lncRNA-mRNA pairs

Click here to Download Table S2

Table S3. GO analyses of differentially expressed mRNAs

Click here to Download Table S3

Table S4. KEGG pathway analyses of differentially expressed mRNAs

Click here to Download Table S4

Table S5. **Clinic characteristics of participants with non-functioning pituitary adenoma or normal pituitary**

Variable	non-functioning pituitary adenoma (n=35)		normal pituitary (n=7)	
	Number	Percentage	Number	Percentage
Age				
<50	13	37.14%	5	71.43%
≥50	22	62.86%	2	28.57%
Sex				
Female	24	68.57%	5	71.43%
Male	11	31.43%	2	28.57%
Tumor size				
<10 mm	3	8.57%	-	-
10-30 mm	15	42.86%	-	-
>30 mm	17	48.57%	-	-
Invasiveness				
Invasive	16	45.71%	-	-
Non-invasive	19	54.29%	-	-

Biology Open • Supplementary information
